# Target trial emulation: harnessing real-world data to evaluate surgery and perioperative care interventions

**DOI:** 10.1093/bjs/znaf182

**Published:** 2025-09-02

**Authors:** Kitty H F Wong, Robert J Hinchliffe

**Affiliations:** Department of Vascular Surgery, Bristol Medical School, University of Bristol, Bristol, UK; Department of Vascular Surgery, Bristol Medical School, University of Bristol, Bristol, UK; Department of Vascular Surgery, North Bristol NHS Trust, Bristol, UK

Randomized controlled trials are the ‘gold-standard’ study design to evaluate the effectiveness of interventions in surgery and perioperative care. Practically, however, RCTs are expensive, complex, and challenging to deliver. As many as 20% of surgical trials are discontinued early due to recruitment and funding issues, representing significant research waste and important ethical considerations over unpublished results^1^. Serious concerns have also been raised about the lack of inclusivity in trials for minority groups. A recent review of 307 RCTs in vascular surgery found significant differences in demographic and clinical factors between patients recruited to vascular RCTs and the real-world population, calling for prompt reflection on the external validity of trial results^2^. RCTs may be impractical in situations where clinical equipoise is challenging or they are practically difficult to conduct, for example in emergency surgery, when there are limited resources, or in the production of timely results in the evaluation of surgical innovations. Consequently, there is a need to develop alternative methods to RCTs.

Recent years have seen an increase in the development and an improvement in the quality of disease-based and surgical registries, which capture procedural-specific data. Linkages to routinely collected clinical and demographic data are now commonplace. Analysis of these ‘real-world data’ (RWD) offer benefits such as large sample sizes containing clinically important subgroups and timely availability of data and represent an opportunity to evaluate the effectiveness of interventions in everyday clinical practice. However, evaluating surgical interventions using observational studies is often limited by data quality and is subject to both measured and unmeasured confounding biases. Consequently, a recent focus has been placed on how to harness RWD such that it can reliably inform guideline development and policy. In the UK, the National Institute of Health and Care Excellence developed the ‘Real-world evidence framework’, encouraging a rigorous standard of evidence through improving data quality and integrating causal inference methodology^3^. RWD has started to play an important role, influencing a number of national technology appraisals, particularly in cost-effectiveness analyses and assessing the early value of new technologies^4^. International regulators, such as the United States Food and Drug Administration^5^ and European Medicines Agency^6^, have also produced guidance publications on the use of RWD to support policy decisions.

The target trial emulation (TTE) framework is an important method recommended in the real-world evidence framework for improving the quality of observational studies and analysis of RWD. TTE describes a systematic approach to the design and analysis of observational data to provide reliable estimates of the effectiveness of interventions. It applies the principles of an RCT addressing the same research question but uses observational data^7^. Investigators emulate an RCT (target trial) by specifying the eligibility criteria, treatment strategy, assignment procedure, follow-up period, outcome measures, the causal contrasts of interest (intention-to-treat or per-protocol effects), and a predetermined robust statistical plan to account for baseline and prognostic factors. Focus is placed on specifying ‘time zero’, representing the point at which eligibility criteria are met, the treatment strategy is assigned, and when follow-up begins. This is analogous to the point of randomization in an RCT. Specifying these components can reduce important biases, such as selection bias and immortal time bias, which occurs when participants are assigned to treated or exposed groups by using information that is observed after the start of follow-up. Additional methods may also be used to account for differences in baseline variables, such as propensity-matching or quasi-experimental methods, to ensure the exposed and non-exposed groups are as similar as possible. The process guide for inferential studies using healthcare data from routine clinical practice to evaluate causal effects of drugs (PRINCIPLED) outlines the process for planning and conducting a study with the TTE approach^8^.

The TTE framework has been compared against RCTs. It has replicated the findings of RCTs with very similar effect estimates in a small number of selected surgical and non-surgical populations, at a fraction of the costs and time required of an equivalent RCT^9^. Recent NIHR-funded studies have proved that it is feasible to perform target trials of selected conditions using RWD in a small group of highly selected surgical conditions and interventions, such as the Emergency Surgery or Not (ESORT) study^10^. However, several challenges were identified, including a lack of specific data variables within the routinely collected RWD to enable stringent specification of some TTE components, including the determination of time zero, intention-to-treat, and accounting for residual confounding. Data quality and availability remain a major limitation to widespread adoption of TTE in formal technology appraisals, and it may be necessary to adapt patient registries going forward to enable robust analysis of RWD. Inherent selection biases of the dataset itself (for example, many registries do not capture patients turned down for surgery) and missing covariates may limit the ability to emulate the ‘ideal’ target trial^9^. TTEs employing a matching approach to compare similarly matched groups can result in the loss of a significant number of participants. Due to the lack of true randomization, intractable confounding and confounding by indication hinder the reliability of results, no matter how well the TTE framework is applied^11^. Further research is required to demonstrate the feasibility of basing healthcare decisions and technology appraisals on observational studies employing TTE, and how to interpret the associated results.

RCTs remain the gold standard in evaluating effectiveness of interventions. However, when these are not feasible or available, TTE using observational RWD may represent a viable alternative to generate valid findings by reducing the risk of important biases, or even as an intermediate step to help justify the need for an RCT. This is especially attractive in areas of surgery where traditional RCTs have faced challenges in recruitment related to equipoise and inclusivity. However, the quality of the available data and intractable confounding remain significant limitations. A standardized approach to study design and reporting guidelines will further improve the quality of target trials^12^. Surgical and perioperative care registries should consider integrating TTE elements into data collection to enable RWD research with robust causal inference methodologies.
